# Body Water During Pregnancy: Physiology, Clinical Significance and Assessment Methods: A Narrative Review

**DOI:** 10.3390/nu18071031

**Published:** 2026-03-24

**Authors:** María Eugenia Flores-Quijano, Reyna Sámano, Edgar Barrientos-Galeana, Hector Borboa-Olivares

**Affiliations:** Coordinación de Nutrición y Bioprogramación, Instituto Nacional de Perinatología, Mexico City 11000, Mexico; ssmr0119@yahoo.com.mx (R.S.); edb.galeana@hotmail.com (E.B.-G.); hector.borboa@inper.gob.mx (H.B.-O.)

**Keywords:** maternal hydration, body fluid compartments, plasma volume expansion, bioelectrical impedance, isotope dilution, maternal physiology

## Abstract

Total body water (TBW) undergoes substantial physiological expansion during pregnancy, reflecting coordinated cardiovascular, renal, and endocrine adaptations required to support maternal metabolism, uteroplacental perfusion, and fetal growth. These changes involve not only an overall increase in body water but also shifts in the distribution of extracellular water (ECW) and intracellular water (ICW), which influence maternal body composition, the interpretation of biochemical biomarkers affected by hemodilution, and pregnancy-related clinical outcomes. Despite its physiological and clinical relevance, the regulation and assessment of body-water compartments during pregnancy remain insufficiently integrated within nutritional and clinical research. This narrative review synthesizes current knowledge on the physiological regulation of TBW and its compartments across gestation and provides a critical analysis of the methodological approaches used to assess body-water distribution in pregnant populations. We review the mechanisms underlying plasma volume expansion, interstitial fluid accumulation, and tissue hydration, and discuss their implications for fetal growth, hypertensive disorders of pregnancy, and gestational diabetes mellitus. We also examine the principles, strengths, and limitations of the main techniques used to assess TBW and body-water compartments during pregnancy. Isotope dilution using stable isotopes (^2^H_2_O and H_2_^18^O) remains the reference method for TBW assessment, whereas bioelectrical impedance-based approaches, including bioelectrical impedance analysis (BIA), bioelectrical impedance spectroscopy (BIS), and bioelectrical impedance vector analysis (BIVA), offer practical alternatives for longitudinal monitoring of fluid redistribution during gestation. By integrating physiological and methodological perspectives, this review provides a framework for understanding body-water dynamics during pregnancy and for selecting appropriate approaches to assess maternal body composition and hydration.

## 1. Introduction

Total body water (TBW) refers to the sum of all water within the body and is broadly divided into two main compartments: intracellular water (ICW), which is contained within cells, and extracellular water (ECW), which is located outside the cellular space [1]. ECW comprises the intravascular compartment (plasma), the interstitial compartment occupying the space between cells, and a smaller fraction of transcellular fluids, including cerebrospinal, synovial, and, during pregnancy, amniotic fluid. Intracellular water reflects the water associated with metabolically active tissues and is closely linked to cell mass and cellular function [2].

In healthy nonpregnant adult women of normal weight, TBW typically accounts for about 55% of total body mass [3]. However, this proportion is not static: it remains relatively stable during early and middle adulthood. Still, it tends to decline to approximately 50% after the age of 60, primarily due to an increase in fat mass and a reduction in lean body mass [4]. Across the menstrual cycle, TBW has been reported to show either minimal variation or modest fluctuations, with slightly higher values during the luteal phase, likely reflecting hormonally mediated changes in fluid retention and distribution [5,6,7,8]. However, these variations are generally small and do not substantially affect clinical assessment of TBW in healthy women when standardized measurement conditions are applied [5].

Pregnancy represents a distinct physiological exception to this relative stability. During gestation, TBW increases by approximately 5–8 L, with the majority of this expansion occurring within the extracellular compartment, including plasma, interstitial fluid, and amniotic fluid [2,9,10]. This progressive increase in body water supports essential maternal and fetal functions. Maternal plasma volume expands by approximately 40–50%, contributing to the maintenance of effective circulatory volume, blood pressure regulation, and enhanced uteroplacental perfusion [11]. Expanded body water also facilitates maternal thermoregulation by increasing skin blood flow [12], while the interstitial compartment supports nutrient, gas, and metabolite exchange between the maternal circulation and tissues [2]. In parallel, amniotic fluid, derived from maternal water turnover and fetal contributions, plays a central role in fetal growth, protection, movement, and thermal regulation throughout pregnancy [13,14].

Accurate assessment of TBW during pregnancy has important clinical and nutritional implications. TBW provides integrative information on maternal hydration status, body composition, and cardiovascular and renal adaptation that cannot be inferred from body weight, body mass index (BMI), gestational weight gain, or anthropometry alone. Deviations in the magnitude or distribution of TBW, particularly disproportionate expansion of extracellular fluid, have been associated with adverse pregnancy outcomes, including hypertensive disorders, impaired placental perfusion, and fetal growth restriction [15,16].

Despite its relevance, the measurement of TBW in pregnancy poses methodological challenges. Reference methods based on isotope dilution using deuterium oxide (^2^H_2_O) or oxygen-18-labeled water (H_2_^18^O) provide highly accurate estimates of TBW but require specialized analytical infrastructure and prolonged equilibration periods, which limit their routine clinical use [10,17]. In contrast, bioelectrical impedance-based techniques, including single- and multifrequency BIA, bioelectrical impedance spectroscopy (BIS), and bioelectrical impedance vector analysis (BIVA), offer practical, non-invasive alternatives that are well suited for longitudinal monitoring across gestation, although their validity depends on physiological assumptions that may be altered during pregnancy [17,18]. Consequently, reported estimates of body-water compartments during pregnancy, including TBW, ECW, ECW/TBW ratios, and plasma volume, should be interpreted in light of the measurement techniques used, as some studies rely on direct determination using isotope dilution methods whereas others derive these values from bioelectrical impedance-based models.

Therefore, this review aims to describe the physiological changes in total body water and its compartments during pregnancy, discuss their clinical and nutritional relevance, and critically review the validated methods currently available to assess TBW and body-water distribution in pregnant women, with emphasis on isotope dilution and bioelectrical impedance-based techniques.

## 2. Methods

This study was conducted as a narrative review examining the physiological regulation of total body water and its compartments during pregnancy, their clinical implications, and the methods used for their assessment. Relevant literature was identified through targeted searches in PubMed/MEDLINE using combinations of terms related to total body water, pregnancy, extracellular and intracellular water, plasma volume expansion, body composition, isotope dilution, and bioelectrical impedance. Additional relevant studies were identified by reviewing the reference lists of key publications as thematic areas were developed. Studies were selected based on conceptual relevance and synthesized narratively, with evidence organized thematically across physiological, clinical, and methodological domains. Given the heterogeneity of study designs, measurement techniques, and outcomes across the literature, as well as the integrative physiological nature of the topic, a narrative approach was considered more appropriate than a formal systematic review. In addition, when numerical estimates of body-water compartments (e.g., TBW, ECW, or ICW) are discussed throughout the review, these values should be interpreted in the context of the measurement techniques used in the original studies, as differences across reports may partly reflect methodological variability between reference methods such as isotope dilution and indirect estimation approaches based on bioelectrical impedance.

## 3. Physiological Mechanisms Modulating Total Body Water and Water Compartments During Pregnancy

During pregnancy, significant changes in the cardiovascular, renal, and hemodynamic systems orchestrate a progressive expansion of TBW and shifts in its distribution across plasma, extracellular, and intracellular compartments.

Early in pregnancy, before placental implantation, systemic vasodilation, mediated primarily by progesterone and relaxin, reduces effective arterial filling and is interpreted as a decrease in circulating volume [19]. Because most circulating blood resides in the venous system, even small reductions in arterial pressure are sensed as underfilling by arterial baroreceptors.

This perceived underfilling initiates and later sustains a neurohumoral response characterized by non-osmotic release of vasopressin and activation of the renin–angiotensin–aldosterone system (RAAS), which promotes systemic vasoconstriction and signals the kidneys to retain sodium and water [20]. In response, the kidneys increase reabsorption in the distal nephron and collecting duct, leading to expansion of ECW, including plasma volume, by approximately 30–50%. Because relatively more water than solute is retained, plasma osmolality and serum sodium fall slightly, producing the characteristic hypervolemic and hypo-osmolar state of pregnancy, in which plasma volume expands disproportionately relative to solute content. Consistent with this adaptive shift, the osmoregulatory threshold for thirst and vasopressin release is reset to a lower plasma osmolarity, thereby facilitating additional water intake and retention [19].

The increase in plasma volume produces a hemodilution-mediated reduction in plasma albumin concentration and a corresponding decrease in colloid oncotic pressure [11,21]. This alteration shifts the balance of Starling forces to favor fluid movement from the intravascular to the interstitial space. Starling forces describe the balance between capillary hydrostatic pressure, which promotes filtration, and plasma colloid oncotic pressure, which retains fluid within the intravascular compartment [22]. During pregnancy, decreased plasma oncotic pressure, combined with elevated capillary hydrostatic pressure from the expanded blood volume, increases the movement of fluid into the interstitial compartment [21].

The interstitial matrix becomes more compliant during pregnancy, partly due to relaxin-mediated remodeling, thereby enhancing its ability to accommodate additional fluid [23]. Experimental data also indicate that renal interstitial hydrostatic pressure is lower in pregnancy, supporting an enhanced capacity for interstitial fluid storage [24]. Only after the extracellular expansion plateaus does intracellular fluid volume gradually increase, driven by osmotic equilibration and fetal and maternal tissue growth, particularly in the breasts, and the pelvic region [11,25]. Consistent with this pattern, the ECW/ICW ratio progressively increases across gestation, reflecting the relatively greater expansion of the extracellular space compared with ICW [25].

Although amniotic fluid is not part of maternal extracellular or intracellular water stores, it constitutes a distinct fluid compartment whose regulation is closely linked to maternal-fetal fluid dynamics, particularly in early gestation [13]. During early pregnancy, amniotic fluid water is derived predominantly from maternal plasma through transmembranous transfer across the amniotic and chorionic membranes, as well as diffusion across the non-keratinized fetal skin, functionally linking amniotic fluid volume to maternal extracellular fluid status [13]. As placental circulation and fetal physiology mature, fetal urine production and pulmonary fluid secretion progressively become the main sources of amniotic fluid, while fetal swallowing and intramembranous absorption regulate fluid removal. Following keratinization of the fetal skin in mid-gestation, amniotic fluid regulation becomes increasingly driven by fetal mechanisms [13], consolidating it as a physiologically distinct water compartment, separate from maternal ECW and ICW. Consequently, amniotic fluid volume has been studied using dedicated approaches, such as direct measurement, dye dilution techniques, and ultrasound-based estimation, rather than maternal body-water assessment methods [26].

In summary, pregnancy is characterized by coordinated changes in extracellular and intracellular water that drive a progressive increase in total body water. Appreciating these physiological shifts is essential for interpreting maternal nutritional and clinical status and for guiding the choice of appropriate methods to assess total body water during pregnancy.

## 4. Clinical and Physiological Importance of Assessing Body Water During Pregnancy

Assessment of TBW and its compartments during pregnancy is fundamental for evaluating maternal nutritional and clinical status, as body water is a central determinant of body composition, hydration, and metabolic function [27,28]. Quantifying TBW provides information that cannot be inferred from body weight, BMI, gestational weight gain, or anthropometry alone. In addition, accurate assessment of body water improves the interpretation of biochemical markers influenced by plasma volume expansion and supports a physiologically grounded evaluation of maternal adaptation across gestation. Importantly, deviations in the magnitude or distribution of TBW from expected physiological patterns have been consistently associated with adverse maternal and perinatal outcomes [27,29].

### 4.1. Body Water, Body Composition, and Nutritional Assessment

From a nutritional and body-composition perspective, TBW measurement is particularly important because the hydration factor (HF), defined as the ratio of TBW to fat-free mass (FFM), changes substantially during pregnancy [27]. HF represents a key parameter linking body water and body composition because it reflects the degree of hydration of fat-free tissues, which underlies the interpretation of body-composition models during pregnancy. HF increases progressively across gestation due to extracellular fluid expansion and increased hydration of maternal and fetal tissues, thereby reducing the density of FFM. Importantly, HF also exhibits marked biological variability, especially in early pregnancy.

Based on the seminal work of Löf and Forsum, in which HF was derived using a body-composition model combining densitometry and TBW measurements within a two-component framework, interindividual biological variability accounts for 73–82% of total HF variability at approximately 14 weeks of gestation, compared with only 0.5–1.7% at 32 weeks, indicating more homogeneous FFM hydration in late pregnancy [27]. In this approach, TBW contributes to the estimation of body fat and, consequently, to the derivation of FFM; therefore, TBW and the resulting FFM estimates are not fully independent. This pattern, later emphasized by Widen and Gallagher [10], implies that two-compartment body-composition models may be particularly imprecise in early gestation, when hydration of fat-free mass shows substantial interindividual variability, and may become progressively biased later in pregnancy as hydration systematically exceeds the non-pregnant reference value of 0.73.

Although gestational-age-specific HF coefficients may partially improve two-compartment models, these estimates still involve methodological interdependence between TBW and FFM and are derived largely from normal-BMI, relatively homogeneous populations and may not be generalizable [10]. Direct measurement of TBW within three- or multi-compartment models therefore offers a more robust approach, reducing reliance on fixed hydration assumptions and strengthening the assessment of maternal nutritional status across pregnancy [30].

### 4.2. Hemodilution and Interpretation of Biochemical Markers

Physiological expansion of total body water during pregnancy has a direct impact on the interpretation of nutritional biomarkers [11,31]. As described above, this expansion is largely driven by a 30–50% increase in plasma volume that exceeds the rise in red cell mass, resulting in hemodilution. Consequently, circulating concentrations of hemoglobin, albumin, and other plasma-soluble biomarkers may appear reduced despite adequate body stores [11,31]. Recognizing these physiological changes is essential when interpreting nutritional biomarkers and comparing values against pregnancy-specific reference ranges and diagnostic cut-offs.

Hemodilution also affects the interpretation of other nutrients measured in plasma. For example, total serum calcium concentration typically decreases during pregnancy, largely due to reduced serum albumin levels secondary to plasma volume expansion, which lowers the albumin-bound fraction of calcium. Importantly, the physiologically active fraction, ionized calcium, remains stable [11].

Overall, understanding the magnitude and timing of pregnancy-related body-water expansion is critical for accurate interpretation of biochemical indicators and strengthens both clinical assessment and research-based evaluation of maternal nutritional status across gestation.

### 4.3. TBW, Plasma Volume Expansion, and Fetal Growth

Adequate expansion of maternal plasma volume is a key physiological adaptation of pregnancy, as it supports uteroplacental perfusion, oxygen and nutrient delivery, and fetal growth. Insufficient plasma volume expansion has long been associated with fetal growth restriction and lower birth weight [11,32].

Several studies, conducted in different populations, have examined the relationship between maternal TBW and birth weight, yielding results that can be interpreted through two complementary, but distinct, physiological mechanisms. On one hand, multiple investigations report a positive association between maternal TBW or fat-free mass and birth weight, consistent with the role of plasma volume expansion in supporting fetal growth. In a U.S. cohort, Butte et al. showed that gestational gains in TBW measured by deuterium dilution were positively correlated with birth weight (r = 0.37, *p* = 0.006) [33]. Similarly, an Italian study using bioelectrical impedance found that a reduction in resistance during the second trimester, reflecting increased TBW and fat-free mass, was independently associated with higher birth weight [18]. Lederman et al. also reported that, at 37 weeks of gestation, each additional liter of maternal body water was associated with an increase of approximately 23 g in birth weight [34]. These findings suggest that greater maternal TBW, which partly reflects plasma volume expansion, may enhance placental perfusion through higher cardiac output and improved uteroplacental blood flow [18,33]. These observations support the concept that maternal TBW may act as an integrative physiological marker of the hemodynamic adaptations required to sustain placental function and fetal growth.

On the other hand, some studies have reported an inverse association between TBW and birth weight, highlighting the importance of maternal nutritional status and body composition. In a study from India, Rush et al. observed that a decrease in TBW between mid and late pregnancy was associated with higher birth weight, but only among rural women [35]. Similarly, in Guatemalan women assessed by bioimpedance, early deposition of dry fat mass (fat) before GW 19, rather than TBW gains, was independently related to fetal growth. The authors proposed that early gestational fat accretion may act as an energy reserve to support the increased metabolic demands of late pregnancy [36].

Taken together, these findings suggest that the relationship between maternal TBW and birth weight is context-dependent and likely reflects the interaction between maternal energy reserves, plasma volume expansion, and overall nutritional status. In women with adequate energy stores, plasma volume expansion and TBW gains may play a dominant role in supporting fetal growth, whereas in nutritionally constrained settings, early fat accumulation may be more critical. Thus, the apparent inconsistencies across studies likely reflect differences in maternal metabolic context rather than contradictory physiological mechanisms.

Differences across studies may also arise from methodological factors, including whether TBW was directly measured using isotope dilution techniques or indirectly estimated using bioelectrical impedance models, as well as from the equations applied to derive body-composition components. Importantly, plasma volume is rarely measured directly in these studies and is generally inferred from changes in total body water or extracellular water compartments. Bioimpedance methods estimate TBW and ECW rather than plasma volume itself; therefore, interpretations regarding plasma volume expansion are indirect.

### 4.4. Hypertensive Disorders of Pregnancy and Fluid Compartment Redistribution

In hypertensive disorders of pregnancy, disproportionate expansion of ECW relative to effective circulating volume is a hallmark feature [1,37]. Although TBW may increase, endothelial dysfunction, increased vascular permeability, and reduced colloid oncotic pressure promote fluid shift from the intravascular to the interstitial space, resulting in reduced effective plasma volume despite overall fluid expansion. This mechanism also underlies the mild, gravity-dependent edema commonly observed in late gestation [1,37].

Hemodynamic and bioimpedance studies indicate that patterns of fluid redistribution differ across hypertensive phenotypes [25]. In early-onset preeclampsia, absolute values of TBW, ECW, and ICW may not differ significantly from uncomplicated pregnancies in some cohorts; however, ECW/ICW ratios are often elevated, reflecting abnormal compartmental distribution rather than true volume overload [25]. This pattern is associated with reduced plasma volume expansion, low cardiac output, increased systemic vascular resistance, and impaired venous capacitance [1,38].

In contrast, late-onset preeclampsia is characterized by higher TBW and ECW, together with elevated ECW/ICW ratios, reflecting fluid overload within a more compliant venous system and relatively preserved or mildly increased cardiac output [25]. Gestational hypertension typically shows a milder phenotype, with modest TBW and ECW expansion and less pronounced alterations in venous capacitance [25,38]. Collectively, these observations indicate that assessment of TBW and its compartmental distribution can help discriminate hypertensive phenotypes and clarify their underlying physiological disturbances.

### 4.5. Metabolic Implications of Intracellular Water and ECW/ICW Ratio

Beyond hemodynamic adaptations, ICW and the ECW/ICW ratio provide insight into maternal metabolic status [39]. ICW is closely related to metabolically active tissues, particularly skeletal muscle, which plays a central role in insulin-mediated glucose disposal. Accordingly, alterations in ICW or a relative expansion of ECW, reflected in a higher ECW/ICW ratio, may signal metabolic vulnerability during pregnancy, especially in women with higher adiposity or insulin resistance [39].

Because adipose tissue contains relatively little ICW, individuals with higher fat mass tend to exhibit elevated ECW/ICW ratios. Adipose tissue has a substantially lower water content than fat-free tissues, containing approximately 10–30% water compared with about 70–73% in fat-free mass, which helps explain why increases in adiposity contribute relatively little to total body water expansion [4,40]. In pregnancy, Xu et al. [39] demonstrated that a higher ECW/ICW ratio is independently associated with increased risk of gestational diabetes mellitus (GDM), particularly among women with higher pre-pregnancy BMI and advanced maternal age. Complementary evidence suggests that greater ICW and fat-free mass are protective against GDM, independent of BMI [39]. However, although early-pregnancy TBW differs between women who later develop GDM and those who do not, TBW does not emerge as an independent predictor after adjustment for confounders, indicating that associations reflect underlying body composition rather than TBW per se [41,42,43].

These findings are consistent with data from non-pregnant populations, where elevated ECW/ICW ratios and reduced ICW are associated with insulin resistance and type 2 diabetes mellitus [44]. Together, they suggest that ECW/ICW ratio may serve as a physiologically meaningful marker of maternal metabolic phenotype during pregnancy.

**Figure 1:**
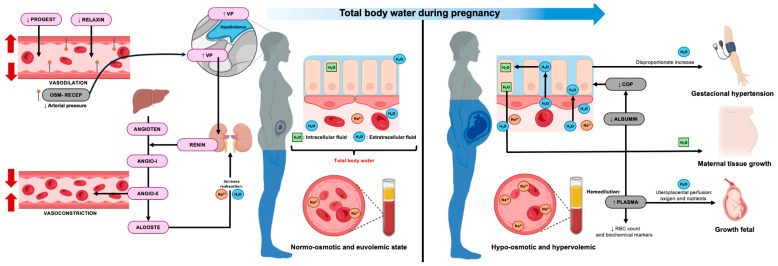
Physiological mechanisms and clinical importance of total body water and fluid compartments during pregnancy. During pregnancy, total body water (TBW) increases as a result of coordinated hormonal, renal, and vascular adaptations. Early vasodilation and neurohormonal activation promote sodium and water retention, leading to plasma volume expansion and a predominant rise in extracellular water (ECW), while intracellular water (ICW) increases more gradually with maternal tissue growth. Expansion of plasma volume exceeds red cell mass, resulting in physiological hemodilution and reduced circulating concentrations of plasma-soluble biomarkers. Reduced colloid oncotic pressure favors interstitial fluid accumulation and mild edema in late gestation. Disproportionate ECW expansion characterizes hypertensive disorders of pregnancy. Overall, these adaptations support uteroplacental perfusion and fetal growth while illustrating the importance of TBW compartmentalization in maternal physiology. The horizontal blue arrow at the top represents the progressive increase in TBW across gestation. VP: vasopressin; PROGEST: progesterone; OSM-RECEP: osmoreceptor; ANGIOTEN: angiotensinogen; ALDOST: aldosterone; COP: colloid osmotic pressure; RBC: red blood cell.

In summary, pregnancy is characterized by profound and dynamic changes in total body water and its compartmental distribution, with important implications for maternal nutrition, metabolic health, hemodynamics, and fetal growth. Accurate assessment of TBW and its compartments improves interpretation of body composition, biochemical markers, and pregnancy outcomes, providing a strong physiological rationale for the careful selection of body-water measurement techniques, which are addressed in the following section. The physiological mechanisms and clinical relevance of total body water and fluid compartment changes during pregnancy are schematically illustrated in Figure 1.

## 5. Methods for Assessing Total Body Water During Pregnancy

A variety of methods are available to quantify TBW, each based on different physical principles and with specific advantages and limitations in pregnant populations [30]. In addition to techniques that estimate TBW, several methods also allow the assessment of ECW and ICW [28], which is particularly relevant in pregnancy because fluid expansion does not occur uniformly across compartments. In this section, we summarize the main techniques used to assess BW and its compartments during pregnancy and discuss their validity, feasibility, and practical implications, as well as considerations for choosing among them.

### 5.1. Dilution-Based Methods

#### 5.1.1. Isotope Dilution for Total Body Water

**Figure 2:**
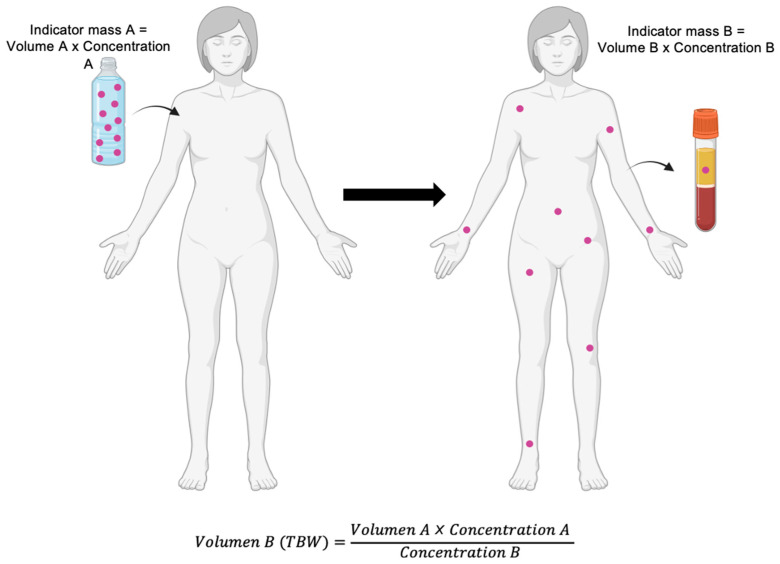
Indicator dilution principle for the estimation of body compartment volume. This method is based on the administration of a known amount of an indicator (mass A = volume A × concentration A), which distributes within the compartment of interest until equilibrium is reached. After uniform dispersion, the final concentration of the indicator is measured in a biological sample (concentration B), allowing calculation of the compartment volume (volume B, e.g., total body water [TBW]) using the relationship volume = indicator mass/measured concentration. For the method to be valid, the indicator must fulfill three fundamental principles: (1) homogeneous distribution throughout the compartment, (2) confinement to the specific compartment being measured, and (3) absence of metabolism or excretion during the measurement period, in addition to being non-toxic. The type of biological sample used for concentration determination depends on the indicator selected and on the feasibility and availability of analytical methods for its subsequent measurement. Purple dots represent the administered tracer, which distributes throughout the body-water compartment after equilibration. The bottle indicates the known administered dose (mass A), whereas the test tube represents the biological sample used to determine the equilibrium concentration (concentration B).

Because water is the only molecular species within the body-water compartment, the dilution principle can be applied to quantify its volume: the size of the compartment equals the amount of tracer administered divided by its equilibrium concentration once uniformly distributed within body water (Figure 2) [3,45]. Isotope-dilution methods operationalize this principle and are regarded as the reference standard for measuring TBW.

Two stable isotopes have been used for TBW assessment during pregnancy: deuterium oxide (^2^H_2_O) and oxygen-18-labeled water (H_2_^18^O) [45]. Both are non-radioactive and considered safe for use in women of childbearing age and during pregnancy when administered at tracer doses [45].

The procedure begins with the collection of a baseline breath, saliva, urine, or blood sample to determine natural isotope abundance (for deuterium, typically ~0.015 atom%). A known oral dose of the isotope is then administered, followed by an equilibration period during which the tracer distributes throughout the body-water compartments. After equilibration, post-dose samples are collected, and isotope enrichment is quantified using various assays, such as isotope-ratio mass spectrometry (IRMS) or Fourier-transform infrared spectroscopy (FTIR) [3,45].

The validity of isotope dilution relies on several methodological assumptions that require particular attention in pregnancy, where fluid dynamics differ from the non-pregnant state [45].

First, the tracer is assumed to distribute exclusively within body water [45]. In practice, a small fraction exchanges with non-aqueous pools, resulting in a dilution space that slightly exceeds true TBW. This exchange is substantially greater for deuterium, which exchanges with non-aqueous hydrogen in proteins and lipids (~4%), than for oxygen-18, which shows more limited exchange with non-aqueous oxygen (~0.7%) [46]. Accordingly, isotope-specific correction factors are routinely applied when calculating TBW.

Second, the tracer isotope is assumed to equilibrate uniformly across body-water compartments [45]. Although isotopic fractionation occurs during evaporative water losses, such as transdermal evaporation and exhaled breath, which are depleted in ^2^H and ^18^O relative to liquid body water, this source of error is minimized under standardized conditions. Protocols therefore recommend avoiding heat exposure and physical activity during the equilibration period, rendering fractionation effects negligible for TBW estimation using urine or saliva samples [3,45].

Third, isotopic equilibration is assumed to be rapid relative to the sampling schedule [45]. In non-pregnant adults, equilibrium is typically achieved within ~2–3 h after oral dosing. During pregnancy, equilibration may be delayed. Using H_2_^18^O, Denne et al. demonstrated that equilibration in pregnant women occurs at approximately 3 h, with near-equilibrium between maternal and amniotic fluid compartments reached after ~5 h. Studies using deuterium oxide suggest that equilibration with amniotic fluid may require slightly longer [47]. The route of isotope administration also influences equilibration time, with intravenous dosing reducing time to equilibrium by approximately one hour compared with oral administration [45]. Consequently, most pregnancy studies, depending on the isotope administration route, allow 4–6 h before post-dose sampling to ensure complete equilibration [35,48,49].

Finally, the method assumes no significant water gain or loss during the equilibration period [45]. Because pregnancy is characterized by increased fluid turnover, participants are typically asked to void their bladder before dosing, minimize food and fluid intake during equilibration, and record any unavoidable intake so it can be accounted for in calculations. When light meals are required, for example, in pregnant participants, these are preferably provided at least one hour after isotope administration, allowing time for tracer absorption and mixing with the body-water pool [3,45].

Both ^2^H_2_O and H_2_^18^O provide accurate (1–2%) estimates of TBW [45]. Oxygen-18 has the theoretical advantage that its dilution space more closely approximates true TBW due to lower non-aqueous exchange. However, this advantage is modest, and the dilution spaces of ^2^H and ^18^O are highly reproducible and mathematically interconvertible with minimal error. In practice, the use of H_2_^18^O is limited by its substantially higher cost and the requirement for IRMS, whereas deuterium can be analyzed using both IRMS and FTIR [45]. For these reasons, deuterium oxide remains the preferred tracer for TBW assessment during pregnancy, balancing accuracy, safety, feasibility, and cost.

Overall, the main limitations of isotope dilution include its cost, need for specialized analytical facilities, participant burden related to repeated sampling, and limited applicability in large epidemiological studies or routine clinical practice. Nevertheless, it remains the reference method against which other TBW assessment techniques are validated.

#### 5.1.2. Dilution Methods for Specific Body-Water Compartments

ECW can be assessed using dilution techniques, most classically with bromide as a tracer, while intracellular water ICW is derived by difference from TBW [45]. Bromide behaves predominantly as an extracellular anion, with a distribution similar to chloride, and therefore distributes mainly within the extracellular fluid space [50]. For this reason, bromide dilution has long been considered a reference method for ECW assessment in non-pregnant adults; however, its application during pregnancy has been limited.

For compartment-specific measurements to be valid, the same methodological assumptions required for TBW assessment, regarding tracer distribution, equilibration, and control of fluid balance, must also be carefully respected [45]. Bromide distributes primarily within the extracellular space but partially penetrates erythrocytes and other cells, resulting in a dilution space approximately 5–10% larger than true ECW, which requires correction [45,50]. In addition, equilibration is relatively slow, requiring approximately 4–6 h under conditions of extracellular fluid expansion, and strict control of posture and fluid intake is needed during the equilibration period [45]. These requirements are particularly challenging in pregnancy, where body water is dynamically redistributed and extracellular volume is physiologically expanded.

Validation studies in pregnant women have demonstrated that bioimpedance-based techniques can capture gestational changes in body-water compartments with reasonable agreement with dilution methods. For example, Van Loan et al. reported that bioimpedance spectroscopy tracked pregnancy-related changes in TBW and ECW with acceptable agreement compared with isotope dilution techniques [51].

Despite these limitations, a small number of studies have applied bromide dilution in pregnant women, primarily to validate non-invasive techniques. In a study by van Loan et al., bromide dilution was used alongside BIS to assess changes in body-water compartments across gestation [51]. The authors reported progressive increases in ECW during pregnancy and demonstrated good agreement between BIS-derived ECW estimates and those obtained by bromide dilution, supporting the validity of impedance-based methods for tracking extracellular fluid expansion during pregnancy [51]. Similarly, Löf et al. evaluated BIS against dilution techniques in healthy pregnant women and found that BIS provided reliable estimates of ECW and ICW changes across gestation, with acceptable agreement at the group level, despite some individual variability [27].

These validation studies indicate that, although bromide dilution is not routinely used in pregnancy, it has played an important role in establishing the physiological plausibility and methodological validity of impedance-based approaches for assessing body-water distribution during gestation. Due to the methodological complexity of bromide dilution, together with the availability of non-invasive alternatives, most pregnancy studies assessing ECW and ICW now rely on bioimpedance-based techniques rather than compartment-specific dilution methods.

### 5.2. Bioelectrical Impedance-Based Methods

Bioelectrical impedance techniques estimate body-water compartments based on the electrical conductive properties of biological tissues. Because body water and dissolved electrolytes conduct electrical current, whereas fat mass and bone are relatively poor conductors, whole-body impedance is closely related to TBW and its distribution between ECW and ICW.

Impedance (Z) represents the opposition of body tissues to the flow of an alternating electrical current and is a vector quantity composed of resistance (R) and reactance (Xc), according to the relationship Z^2^ = R^2^ + Xc^2^ [52]. Resistance reflects the conductive pathway through ionic solutions and is inversely related to body water volume. Reactance arises from the capacitive properties of cell membranes and tissue interfaces, that is, their ability to temporarily store electrical charge and delay current flow, providing indirect information on cell mass and membrane integrity [52].

#### 5.2.1. Measurement Principles and Device Configurations

Impedance measurements are traditionally obtained using a tetrapolar whole-body configuration, in which a low-intensity alternating current is injected through two surface electrodes (i.e., left hand and foot), while voltage drop is measured by a separate pair of electrodes placed proximally on the same side of the body (left wrist and ankle) [53]. This whole-body BIA model assumes that the human body behaves as a single, homogeneous cylindrical conductor. Under this assumption, electrical resistance is directly proportional to conductor length and inversely proportional to cross-sectional area, allowing estimation of conductive volume, and thus TBW, from the ratio of height squared to resistance. However, because body geometry is heterogeneous, particularly due to the large, low-resistance trunk, this simplification introduces systematic error [52].

**Figure 3:**
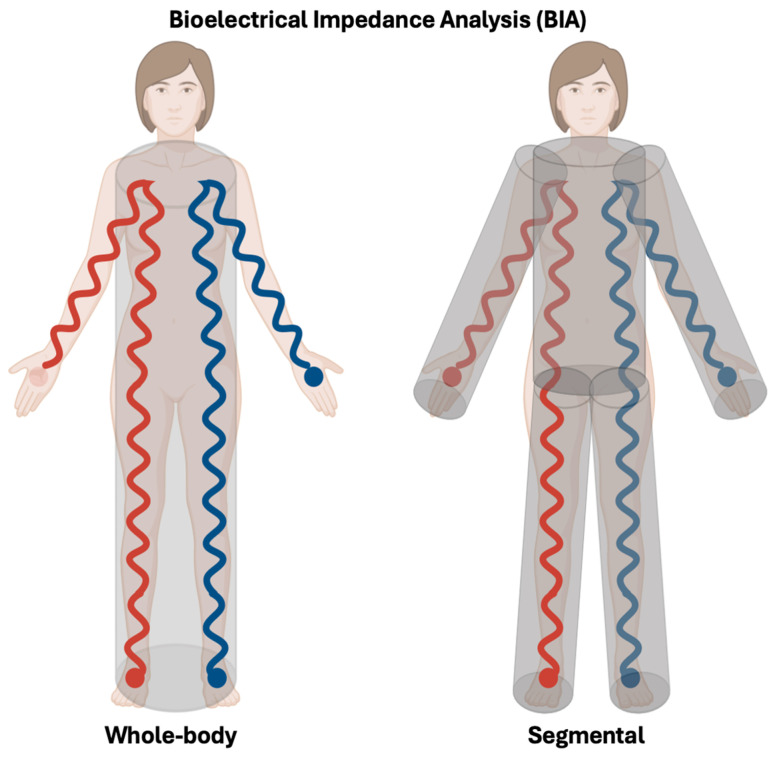
Whole-body versus segmental bioelectrical impedance analysis (BIA). Schematic representation of the traditional tetrapolar whole-body BIA configuration (**left**), in which a low-intensity alternating current is injected through distal electrodes placed on the hand and foot, while voltage drop is measured proximally at the wrist and ankle, assuming the body behaves as a single homogeneous cylindrical conductor. This model estimates TBW from the relationship between body length and electrical resistance. In contrast, segmental BIA (**right**) models the body as five interconnected cylinders (four limbs and the trunk) and independently measures impedance in each segment using multiple tactile electrodes. This approach accounts for regional differences in body geometry and resistance, improving the estimation of TBW and fluid distribution, particularly in conditions characterized by heterogeneous fluid shifts such as pregnancy. Red and blue lines represent the pathway of the applied alternating electrical current through the body between electrodes.

More recent devices address this limitation through segmental BIA, modeling the body as five interconnected cylinders (four limbs and the trunk) and independently measuring impedance in each segment (Figure 3). Modern systems typically use eight tactile electrodes (two per hand and foot), enabling direct segmental analysis without adhesive electrodes. This approach improves estimation of TBW and fluid distribution, especially under physiological conditions such as pregnancy, where regional fluid shifts and trunk enlargement are pronounced [52,53].

#### 5.2.2. Frequency-Dependent Assessment of TBW, ECW and ICW

Single-frequency BIA uses a 50 kHz current that passes through both extracellular and intracellular fluids, providing an indirect estimate of TBW based on population-derived prediction equations [54]. Importantly, single-frequency devices operating at 50 kHz may differ in their technical configuration. Phase-sensitive instruments measure both resistance and reactance and allow calculation of phase angle, whereas non-phase-sensitive devices measure resistance only [54]. These technical differences can influence the estimation of body-water compartments and should therefore be considered when comparing results across studies.

In contrast, multifrequency BIA (MF-BIA) measures impedance at several discrete frequencies [15,54]. This approach is based on the principle that low-frequency currents are largely confined to the extracellular space, as they cannot penetrate cell membranes, allowing estimation of ECW. At higher frequencies, current flows through the whole-body water and reflects TBW. ICW is subsequently derived by difference (TBW − ECW), using empirical prediction equations [15,54].

Bioelectrical impedance spectroscopy (BIS) further extends this concept by measuring impedance across a continuous frequency spectrum (approximately 5–1000 kHz) and applying a biophysical Cole–Cole model to describe tissue electrical behavior. From this model, resistance at zero frequency (R_0_), reflecting ECW, and resistance at infinite frequency (R∞), reflecting TBW, are derived, allowing ICW to be calculated by difference [54]. This approach is often described as a “mixed theory” method because it combines fundamental electrical models of tissue behavior with empirical assumptions about body geometry and fluid distribution to derive physiological compartments.

Validation studies comparing BIS with isotope dilution techniques indicate that impedance spectroscopy can detect gestational changes in fluid compartments with acceptable agreement. For example, Van Loan et al. reported that BIS-derived estimates of TBW and ECW showed good agreement with isotope dilution measurements and were able to track pregnancy-related increases in body-water compartments across gestation [51]. These findings suggest that BIS can capture the longitudinal expansion of maternal body water during pregnancy, supporting its use for monitoring physiological fluid redistribution.

These frequency-based approaches are particularly relevant in pregnancy, where physiological expansion of body water occurs preferentially in the extracellular compartment and does not proceed uniformly across gestation. Nevertheless, the validity of BIA and BIS relies on several underlying assumptions that require careful consideration during pregnancy [15].

First, the body is approximated as a series of uniform cylindrical conductors, and impedance is interpreted using prediction equations that scale measurements to height and body weight [15,35]. During pregnancy, however, abdominal enlargement, changes in limb fluid distribution, and progressive gestational weight gain alter body geometry and segmental fluid distribution, potentially affecting current pathways.

Second, impedance models assume constant resistivity coefficients for intra- and extracellular fluids; however, pregnancy involves changes in plasma volume, electrolyte distribution, and interstitial hydration that may modify tissue resistivity independently of true changes in body composition [15,35].

Third, impedance measurements assume a stable distribution of body water at the time of assessment; posture, venous pooling, recent physical activity, and fluid intake can therefore influence results, particularly in late gestation [15,35].

For these reasons, standardized measurement conditions are essential to improve the reliability of BIA and BIS during pregnancy. Assessments should be performed at same time of day, adequate rest before measurement, avoidance of food or large fluid intake for at least 4 h, and bladder emptying prior to testing [15]. These precautions are particularly important in pregnancy, when rapid shifts in fluid distribution, venous pooling, and edema may otherwise introduce variability unrelated to true changes in body-water compartments.

Despite these limitations, BIA and BIS offer important practical advantages in pregnancy research and clinical settings. They are non-invasive, rapid, portable, and suitable for repeated measurements across gestation. Although absolute estimates of TBW, ECW and ICW are less accurate than isotope dilution [28,35], impedance-based techniques are well-suited for longitudinal assessment of fluid redistribution and for distinguishing physiological from pathological patterns of water expansion, such as those observed in hypertensive disorders of pregnancy [1,25,38]. Consequently, BIA and BIS represent the most widely used methods for assessing body-water compartments in pregnant populations, complementing isotope dilution approaches.

#### 5.2.3. Bioelectrical Impedance Vector Analysis (BIVA)

In addition to conventional BIA and BIS approaches, bioelectrical impedance vector analysis (BIVA) provides an alternative, equation-free framework for interpreting impedance measurements to assess hydration status, body cell mass, and cell membrane integrity, which are indicators of healthy physical function or pathology [55].

In simple terms, instead of converting resistance (R) and reactance (Xc) into estimates of body-water volumes using prediction equations, BIVA plots these raw measurements, standardized for height, as a vector in a two-dimensional graph. The position and length of this vector reflect the individual’s hydration status and body cell mass relative to reference populations.

In BIVA, resistance normalized by height (R/H) is plotted on the x-axis and reactance normalized by height (Xc/H) on the y-axis. Vector length is primarily influenced by total body water, with shorter vectors indicating greater hydration and longer vectors suggesting relative dehydration. Vector direction, reflected by changes in Xc/H, is related to cell membrane integrity and body cell mass. Reference tolerance ellipses derived from healthy populations are used to interpret whether an individual’s vector indicates normal hydration, fluid overload, or reduced cellular mass [55,56]. These ellipses represent the statistical distribution of impedance vectors obtained from population-based samples and provide a graphical framework to classify individual vectors relative to expected physiological ranges.

**Figure 4:**
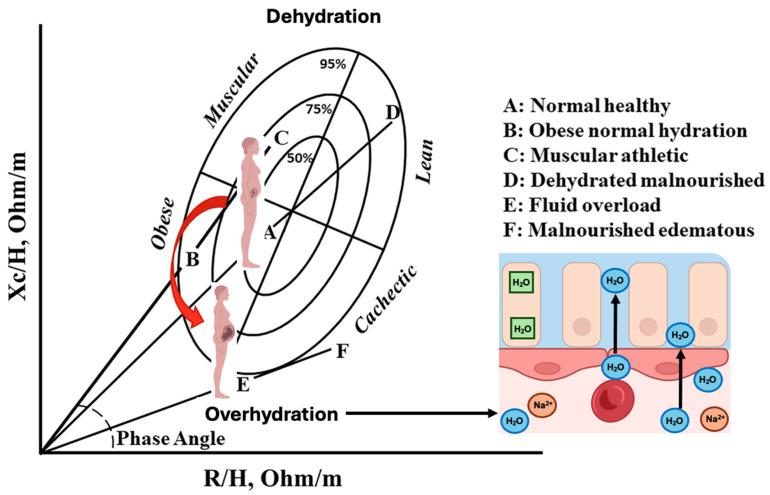
Bioelectrical impedance vector analysis (BIVA) and physiological expansion of total body water during pregnancy. BIVA provides an equation-free framework to assess hydration status and body cell mass by plotting resistance (R) and reactance (Xc). During normal pregnancy, coordinated hormonal, renal, and vascular adaptations promote progressive TBW expansion, predominantly through an increase in extracellular water. This physiological process results in a characteristic shortening and downward displacement of the impedance vector from A (normal healthy, early pregnancy) toward E (late pregnancy), reflecting plasma volume expansion, hemodilution, and mild interstitial fluid accumulation. This trajectory represents the normal course of gestation and distinguishes physiological hydration changes from pathological fluid overload. The red arrow indicates the direction of the impedance vector shift across gestation, from normal early pregnancy toward increased hydration and extracellular fluid expansion in later stages. Adapted and edited from Lukaski, 2023 [56].

A key advantage of BIVA is that it does not rely on assumptions about constant tissue hydration, body geometry, or stable resistivity coefficients. This is particularly relevant during pregnancy, where progressive plasma-volume expansion, interstitial fluid accumulation, and changes in body shape violate many of the assumptions underlying conventional BIA and BIS models (Figure 4).

Several studies have applied BIVA during pregnancy to describe physiological changes in hydration and body composition, in healthy women (see Table 1) [49,57,58,59]. Longitudinal and repeated cross-sectional studies consistently report a progressive reduction in resistance and reactance normalized by height (R/H and Xc/H), leading to vector shortening across gestation, which reflects the expected increase in total body water and overall hydration as pregnancy advances [49,58,59]. Importantly, in uncomplicated pregnancies these vector shifts frequently occur without significant changes in phase angle, suggesting that extracellular and intracellular water expand proportionally and that the ECW/ICW ratio may remain relatively stable despite marked increases in total body water [58]. Evidence from one study comparing singleton and twin pregnancies indicates that greater fluid expansion may be accompanied by lower phase angle values, consistent with a relatively larger extracellular fluid contribution [57]. Notably, the studies summarized here have focused primarily on uncomplicated pregnancies. Although preliminary evidence suggests that abnormal vector displacement may reflect fluid imbalance associated with pregnancy complications, such as hypertensive disorders, the available data remain limited. Therefore, the potential application of bioelectrical impedance vector analysis to pregnancy-related conditions such as hypertensive disorders or gestational diabetes warrants further investigation.

Importantly, BIVA does not provide quantitative estimates of TBW, ECW, or ICW and should therefore be considered complementary rather than alternative to isotope dilution or compartmental impedance techniques. Its main value lies in longitudinal monitoring, pattern recognition, and situations where prediction equations may be unreliable, such as late pregnancy or in the presence of edema.

Future work should focus on strengthening the role of bioelectrical impedance-based approaches in the assessment of maternal fluid dynamics during pregnancy. This includes the development of pregnancy-specific reference patterns for impedance-derived parameters, as well as longitudinal studies that further examine their agreement with reference methods across gestation. Refinements in modeling approaches that better account for gestational changes in body geometry and fluid distribution may also improve the physiological interpretation of impedance measurements. In addition, integrating impedance-derived indicators with clinical and metabolic markers could help clarify their potential role in identifying early physiological patterns associated with pregnancy complications.

### 5.3. Comparison of Methods for Assessing Body Water During Pregnancy

Given the methodological diversity and pregnancy-specific constraints of available techniques, a comparative synthesis is useful to highlight their respective principles, strengths, and limitations. Table 2 summarizes the main methods used to assess body water during pregnancy, including isotope dilution, compartment-specific dilution approaches, and bioelectrical impedance-based techniques, with emphasis on accuracy, safety, feasibility, and applicability across gestation.

## 6. Implications for Nutritional and Clinical Assessment of Body Water During Pregnancy

This section synthesizes the main physiological and methodological insights discussed throughout the review and highlights their implications for nutritional and clinical assessment of body water during pregnancy. In summary, accurate assessment of body water during pregnancy is important for understanding maternal nutritional status, physiological fluid adaptation, and the interpretation of biochemical markers affected by plasma volume expansion. Isotope dilution remains the reference method for quantifying total body water, providing a robust physiological benchmark, while bioelectrical impedance-based techniques offer practical advantages for longitudinal monitoring and large-scale studies.

Frequency-based impedance methods, including multifrequency BIA, BIS, and BIVA, are particularly informative for characterizing changes in fluid distribution between extracellular and intracellular compartments, which are central to pregnancy-related adaptations and pathophysiology. However, their validity depends on assumptions that may be challenged by gestational changes in body geometry, resistivity, and fluid compartmentalization. Method selection should therefore be guided by the specific research or clinical question, gestational stage, and the balance between physiological accuracy, feasibility, and interpretability, rather than by a single universal approach.

A strength of this review lies in the integration of physiological mechanisms, clinical implications, and methodological considerations related to body-water assessment during pregnancy. At the same time, as with any narrative synthesis, the selection and interpretation of studies depend on conceptual relevance rather than on a predefined systematic protocol, which represents an inherent limitation of this type of review.

Future research should continue to advance the integration of physiological and methodological approaches to the study of body-water dynamics during pregnancy. In particular, longitudinal designs that link changes in fluid compartments with maternal and fetal outcomes will be essential to strengthen the clinical relevance of body-water assessment. Such efforts will help refine the interpretation of body-water measurements and support their translation into both research and clinical practice.

## 7. Conclusions

Accurate characterization of body-water dynamics during pregnancy remains essential for understanding maternal physiological adaptation and for interpreting body composition and biomarkers influenced by hemodilution. Pregnancy represents a unique biological context in which coordinated cardiovascular, renal, and endocrine changes drive substantial expansion and redistribution of total body water across gestation. Integrating current knowledge on these physiological mechanisms with methodological considerations for assessing body-water compartments is therefore critical when evaluating maternal nutritional status and metabolic health during pregnancy. Continued refinement of measurement approaches and improved characterization of body-water dynamics across gestation will further strengthen both research and clinical interpretation in this field.

## Figures and Tables

**Table 1:** Summary of studies applying BIVA to evaluate hydration during pregnancy.

Author (Country, Year)	Study Objective	Study Design and Sample	Main BIVA Findings	Key Interpretation/Comments
Choi et al. (Korea, 2025) [57]	To compare maternal body composition assessed by BIA/BIVA between singleton and twin pregnancies	Cross-sectional study; 21 pregnant women (>24 gestational weeks): 9 singleton and 12 twin pregnancies	Phase angle was significantly lower in twin compared with singleton pregnancies (5.1° vs. 6.2°, *p* = 0.007)	A lower phase angle suggests altered cellular status and fluid distribution. Twin pregnancies likely exhibit a larger expansion of body water, particularly ECW, contributing to reduced phase angle values.
Moroni et al. (Italy, 2021) [58]	To characterize pregnancy-related changes in body composition using BIVA	Longitudinal (*n* = 12; assessed at 11–15 and 28–32 weeks) and repeated cross-sectional design (different women at 12–13 and 30–31 weeks)	Both longitudinal and cross-sectional analyses showed significant reductions in R/H, Xc/H, and Z/H (*p* < 0.01), with progressive vector shortening	Vector shortening reflects increasing TBW and hydration across gestation. Phase angle remained unchanged, suggesting that ECW and ICW increased proportionally and the ECW/ICW ratio remained relatively stable in healthy pregnancies.
Rodríguez- Atristain et al. (Mexico, 2016) [59]	To describe and compare trimester-specific changes in maternal body composition using BIVA	Repeated cross-sectional study: first trimester (n = 4), second (n = 21), third (n = 40)	Progressive decrease in R/H and Xc/H across pregnancy. Mean ± SD R/H: 385 ± 62.0, 373.6 ± 59.3, 341.9 ± 49.6; Xc/H: 39.3 ± 3.7, 39.2 ± 6.7, 34.4 ± 6.6 (first, second, third trimester). Significant differences between second and third trimesters (*p* < 0.05)	Vector shortening and leftward displacement within tolerance ellipses indicate increased hydration and changes in soft tissue composition during pregnancy.
Lukaski et al. (USA, 2007) [49]	To evaluate whether BIVA reflect changes in TBW and hydration during pregnancy and postpartum	Longitudinal study; women assessed at 14, 26, and 36–38 gestational weeks	Significant reductions in R/H (from 361 ± 10 to 318 ± 10 Ω/m, *p* < 0.05) and Xc/H (from 44 ± 1 to 36 ± 1 Ω/m, *p* < 0.05) across gestation	Changes in impedance vectors paralleled increases in TBW and hydration, supporting BIVA as a qualitative tool for monitoring pregnancy-related fluid changes.

BIA: Bioelectrical impedance analysis BIVA: bioelectrical impedance vector analysis.

**Table 2:** Comparative Overview of Methods for Assessing Body Water during Pregnancy.

Method	What It Measures (Outputs)	Core Principle	Typical Accuracy/Role	Pregnancy Feasibility & Safety	Main Advantages	Main Limitations/Pregnancy-Specific Issues
Isotope dilution—Deuterium (^2^H_2_O)	TBW	Dilution principle: TBW = dose/equilibrium enrichment (with isotope-specific corrections)	Reference method for TBW	High feasibility in research, safe at tracer doses	High validity; field-friendly sampling (saliva/urine); can be repeated longitudinally	Needs lab analysis (IRMS or FTIR), cost/logistics; requires standardized conditions; pregnancy may delay equilibration → often longer equilibration windows
Isotope dilution—Oxygen-18 (H_2_^18^O)	TBW	Same dilution principle	Comparable accuracy to ^2^H_2_O; dilution space closer to TBW (smaller non-aqueous exchange)	Safe at tracer doses; less used due to cost	Strong theoretical fit to TBW; widely accepted in physiology	Typically requires IRMS, labeled water cost higher; limited availability and field feasibility
Dilution for ECW—Bromide	ECW directly; ICW = TBW − ECW (if TBW measured)	Extracellular tracer distribution (bromide behaves similarly to chloride)	Reference-type method for ECW in non-pregnant adults	Not routinely used in pregnancy	Conceptually direct ECW measurement	Slow equilibration; posture/fluid intake control crucial; bromide dilution space overestimates ECW (cell penetration + distribution differences) → needs corrections; limited pregnancy data/feasibility
Single-frequency BIA (SF-BIA)	TBW	Measures impedance at one frequency (commonly 50 kHz) and applies prediction equations	Useful for trends; absolute compartment accuracy depends on equation validity	Very feasible, safe, fast	Cheap, portable; good for repeated measures	Heavily equation-dependent; pregnancy changes geometry/resistivity; trunk + edema can bias.
Multi-frequency BIA (MF-BIA)	TBW + ECW, ICW by difference	Measures impedance at multiple discrete frequencies (low freq ≈ ECW pathway; high freq ≈ TBW pathway), then uses empirical models/equations	Better compartment inference than SF-BIA	Very feasible in pregnancy	More informative than SF-BIA for fluid redistribution	Still equation/model dependent; pregnancy-specific calibration often lacking; posture/edema matters
Bioimpedance spectroscopy (BIS)	TBW, ECW, ICW (model-based), plus parameters from spectral fit	Fits impedance over a spectrum (e.g., 5–1000 kHz) using models (e.g., Cole–Cole) and mixture theory	Strong for longitudinal tracking; compartment estimates depend on model/inputs	Very feasible in pregnancy research	Rich frequency information; can better separate ECW vs. ICW than SF-BIA; practical for cohorts	Assumptions: shape factor/geometry, resistivity coefficients, current pathways; pregnancy alters trunk geometry and fluid distribution; device/software not interchangeable
Segmental impedance (8-point/hand-foot + segment models) (often SF or MF, sometimes BIS)	Segment TBW/ECW surrogates; improves whole-body estimates	Treats body as multiple cylinders/segments (limbs + trunk) rather than single cylinder	Often improves robustness vs. whole-body-only models	Very feasible; common in modern devices	Better handles heterogeneity of trunk vs. limbs; can reduce systematic bias	Still depends on device algorithms; segmental assumptions can still be challenged by pregnancy trunk expansion
BIVA (vector analysis)	Qualitative hydration/cellular status (vector position/length), not TBW volumes	Plots R/H vs. Xc/H as a vector; interprets vs. reference ellipses	Not a TBW “measurement”; a complementary interpretation tool	Very feasible; attractive in pregnancy where equations break	Equation-free; less sensitive to some violated assumptions; good for longitudinal patterning	Does not output TBW/ECW/ICW volumes; interpretation depends on reference sets; pregnancy-specific reference ellipses often limited

TBW: Total body water; FFM: Fat-free mass; IRMS: isotope-ratio mass spectrometry; FTIR: Fourier-transform infrared spectroscopy ECW: Extracellular water; ICW: Intracellular water.

## Data Availability

No new data were created or analyzed in this study.
